# Solid Lipid Nanoparticles Incorporated with Retinol and Pentapeptide-18—Optimization, Characterization, and Cosmetic Application

**DOI:** 10.3390/ijms251810078

**Published:** 2024-09-19

**Authors:** Małgorzata Pawłowska, Marta Marzec, Waldemar Jankowiak, Izabela Nowak

**Affiliations:** 1 Department of Applied Chemistry, Faculty of Chemistry, Adam Mickiewicz University, Poznań, Uniwersytetu Poznańskiego 8, 61-614 Poznan, Poland; malgorzata.pawlowska@amu.edu.pl; 2 Dottore Polska Sp. z o.o., Margonińska 22, 60-425 Poznan, Poland; klinika@kec.pl

**Keywords:** solid lipid nanoparticles, retinol, oligopeptide, high shear homogenization, optimization, stability

## Abstract

Solid lipid nanoparticles (SLNs) incorporated with retinol and oligopeptide can have a full spectrum of effects on the skin as a compatible combination of ingredients with broad anti-aging properties. The research’s main objective was to ensure the stability of lipid nanocarriers containing retinol and peptide due to the planned use of this dispersion as a cosmetic raw material. To confirm the effectiveness of method optimization (high shear homogenization, HSH) and proper selection of substrates, SLN dispersions were obtained in three combinations: 1—non-incorporated SLNs; 2—SLNs containing only retinol; 3—SLNs containing retinol and pentapeptide-18; these were then stored at different temperatures (4, 25, 45 °C) for 4 weeks. The desired values of the physicochemical parameters of the optimized dispersion of lipid nanoparticles incorporated with retinol and oligopeptide over the required storage period were confirmed: mean particle size (Z-Ave) = 134.7 ± 0.3 nm; polydispersity index (PDI) = 0.269 ± 0.017 [−]; zeta potential (ZP) = 42.7 ± 1.2 mV (after 4 weeks at 25 °C). The results confirmed the proper selection of the SLN production method and the effectiveness of the optimization performed. The possibility of using the obtained raw material as an ingredient in cosmetic products with anti-aging properties was indicated.

## 1. Introduction

Systems for the delivery of active substances to the skin are a subject under constant development and improvement processes. Numerous studies carried out in the pharmaceutical and cosmetic industries have contributed to significant advances in the formulation of pharmaceutical and cosmetic products containing encapsulated bioactive substances. However, the physiology and characteristic structure of the skin, which acts as a natural barrier against the external environment, still limit the effectiveness of therapeutics and skin care, causing difficulties in the penetration and absorption of active substances [1]. In the process of overcoming the epidermal barrier and facilitating the penetration of active substances, different types of mechanisms are involved, such as intercellular diffusion, active transport, and carrier-mediated transport [2,3,4]. The transport of bioactive substances can take place through three routes: through the structures of the epidermis, through the outlets of sweat glands, and through hair follicles and sebaceous glands [5]. The effectiveness of the last three ways is less significant due to the small total area that these structures occupy on the skin’s surface (0.1%) [3]. However, in the case of large polar molecules and ionic compounds, which have difficulty penetrating the epidermal layers, these routes play an important role [3]. The route through the appendages of the skin can be used to transport active substances with the participation of appropriately selected lipid carriers, such as solid lipid nanoparticles (SLNs).

In the case of retinol, transport using skin appendages is the primary route of penetration into the deeper layers of the skin [6]. Vitamin A is the first vitamin approved by the U.S. Food and Drug Administration (FDA) as an anti-aging agent that affects the appearance and condition of the skin and exhibits anti-aging activity [7]. Retinoids are an attractive group of compounds with a broad spectrum of effects on the skin. They regulate numerous physiological processes, such as epidermal cell growth, the differentiation of the keratinocytes, and sebaceous gland function. In addition, they exhibit brightening properties, contributing to the proper distribution of melanocytes within the epidermis, and most importantly, they affect the synthesis of collagen and elastin, so they have anti-aging and anti-wrinkle effects on the skin [8,9]. Retinol, as a fat-soluble substance, can penetrate through the epidermis and, to a small extent, into the dermis. For effective operation, it is important to increase its penetration rate [7]. The incorporation of retinoids into the SLN significantly influences the controlled and prolonged release of the substance in the skin and achieves a high penetration rate [8]. Ferreira et al. [6] also point to this process minimizing the side effects of retinoids (in an encapsulated form) including erythema reaction due to irritation or the degree of epidermal desquamation.

On the other hand, peptides—compounds of hydrophilic nature—do not exhibit the possibility of passive absorption through the skin [2]; hence, the availability of delivery methods for peptide compounds is quite limited and constantly a topic of scientific interest. The history of the use of peptides in skin care dates back to the 1980s [10], but there was a particular surge of interest in these compounds at the beginning of the 21st century when the activity of peptides and their involvement in numerous skin processes were recognized [11]. The safety of the substances has been confirmed by the Scientific Committee on Consumer Safety (SCCS) [12] and the FDA in its Cosmetic Ingredient Review (CIR). Peptides, as bioactive compounds, have a multidirectional effect on skin conditions. They exhibit anti-wrinkle and anti-aging effects by inhibiting the enzymatic activities of hyaluronidase, collagenase, and elastase. In addition, they regulate the activity of tyrosinase responsible for the formation of hyperpigmentation [13,14]. Currently, special attention is paid to peptide compounds of higher molecular mass [2]. The use of solid lipid nanoparticles as transdermal carriers for peptide compounds provides them with protection from enzymatic degradation, increases the bioavailability of this active substance, and reduces the likelihood of side effects [15].

Pentapeptide-18 (Tyr-D-Ala-Gly-Phe-Leu) belongs to the group of neurotransmitter peptides. This neuropeptide is associated with the SNAP-25 receptor protein (SyNaptosome-Associated Protein, 25 kDa), which is involved in the release of acetylcholine and the formation of the SNARE (SNAP receptors—Soluble N-ethylmaleimide sensitive factor Attachment protein REceptor) complex. Acetylcholine, responsible for causing muscle contractions and the formation of facial wrinkles, may negatively affect the condition of the skin. Pentapeptide-18 affects receptors at neuromuscular synapses acting like enkephalins. By binding to the receptors of enkephalins, it closes calcium channels and reduces the release of acetylcholine. Clinical studies have unequivocally confirmed the anti-aging and anti-wrinkle effects of the said oligopeptide on skin [16,17].

The use of SLN is a promising way to deliver active ingredients in a cosmetic product due to several advantages that they show over traditional methods [18]: (i) the protection of the incorporated active ingredients (e.g., retinol) from chemical degradation [19]; (ii) the possibility of the controlled and prolonged release of the active ingredient [5,20]; (iii) increasing the stability of the introduced bioactive substances [20]; (iv) the increased absorption of substances across the epidermal barrier and improved depth of penetration through the skin [5]; (v) the occlusive properties of the lipid carriers themselves, which result in reduced transepidermal water loss (TEWL) and have a beneficial effect on skin hydration levels [5,19]. The lack of toxicity and biodegradability of solid lipid nanoparticles are perfectly in line with the trend of sustainable cosmetics and green chemistry. In addition, due to its efficiency, the carrier-based method allows for the use of fewer active ingredients while maintaining effective therapeutic effects.

The purpose of the presented research was to develop a method for obtaining solid lipid nanoparticles containing retinol and an oligopeptide (Tyr-D-Ala-Gly-Phe-Leu). A detailed optimization of the qualitative composition of lipid nanoparticle dispersions was performed, taking into account, among other things, the type of solid lipid, the surfactant, and the presence of liquid lipid in the lipid matrix. The stability of the obtained SLNs was a basic assumption for the optimization of the method since the designed dispersion was ultimately one of the key components of the cosmetic formulation. Moreover, the additive effect of the oligopeptide and retinol (contained in the cosmetic product formulation) created the possibility of providing effective anti-aging therapy in line with the trend of skin minimalism.

## 2. Results and Discussion

### 2.1. Optimization of the Qualitative Composition of Lipid Nanoparticle Dispersions

#### 2.1.1. Lipid Screening—Selection of the Solid Lipid

Lipids, as non-polar compounds, are components of lipid nanoparticle dispersions at levels of 0.1–30.0 wt.% [21]. Moreover, lipids such as mono-, di- or triglycerides of medium- or long-chain fatty acids are, in fact, components of most available emulsion-like cosmetic formulations [22]. The selection of a solid lipid began with a choice of four lipid compounds most commonly used in the formulation of cosmetic products, namely mono- (glyceryl monostearate—Imwitor^®^ 900 K) and diglycerides (glyceryl dibehenate—Compritol^®^ 888 ATO, glyceryl distearate—Precirol^®^ ATO 5) of long-chain fatty acids and mixtures thereof (Witepsol^®^ H15). The selected solid lipid had to be compatible with the incorporated lipophilic active compounds—in this case, retinol—since it is the properly formed lipid matrix that is crucial for the stability of solid lipid nanoparticles [23]. What is more, the solid lipids selected for optimization were safe and commonly used lipid compounds, which, at the same time, ensured production continuity for further commercial implementation. In the course of the experiment conducted on the selection of the solid lipid, Witepsol^®^ H15 and Precirol^®^ ATO 5 were excluded from further testing after the first hour of observation following the solidification of the system (Table 1). These two lipids showed incompatibility with retinol after a preliminary organoleptic evaluation—the resulting mixtures were heterogeneous with irregularities on the surface of the system, which was interpreted as the immiscibility of retinol with the above-mentioned two solid lipids. After an assumed time of 72 h, the quality and compatibility of the remaining systems were evaluated. Two solid lipids, Imwitor^®^ 900 K and Compritol^®^ 888 ATO, were qualified for further optimization of the composition of solid lipid nanoparticles.

The selected lipids Imwitor^®^ 900 K (1.0 wt.%) and Compritol^®^ 888 ATO (1.0 wt.%) were used to produce lipid nanoparticle dispersions. Physicochemical parameters were determined for the SLN samples, differing in the type of solid lipid used. The test was carried out on the day that the dispersion was obtained (D0). Due to the unsatisfactory parameters of the sample containing Compritol^®^ (Z-Ave = 560.2 ± 7.4 nm; PDl = 0.563 ± 0.040; ZP = 45.7 mV ± 0.4), testing after 14 days (D14) was repeated for the formulation containing Imwitor^®^ only. The purpose of the retest was to confirm the validity of the choice of solid lipid. The dispersion in which Imwitor^®^ was used in the formulation was characterized by the following values for the determined parameters: Z-Ave = 207.4 ± 1.1 nm; PDl = 0.446 ± 0.015; ZP = 39.7 ± 0.2 mV (on day 0). The test repeated on day 14 indicated a decrease in the values of individual parameters as follows: by 16% for Z-Ave = 174.4 ± 0.8 nm; by 17% for PDl = 0.369 ± 0.026; and by 3% if ZP = 38.7 ± 0.3 mV was considered.

For the SLN samples whose matrixes were based on Imwitor^®^, the most favorable results were observed, given the assumption made regarding the expected size of the lipid structures tested (Z-Ave < 300 nm). This lipid was selected as a solid lipid suitable for the formation of the lipid matrixes of SLNs. The stability of the optimized nanoparticles was confirmed by test results after 14 days of storage at 25 °C. According to Almeida [15], the selection of a suitable solid lipid in the lipid matrix can affect the release efficiency of the active substance, especially protein substances, which are in the research area of this paper. The presented comparison of the results obtained for samples based on Compritol^®^ and Imwitor^®^ indicated that more favorable effects of delivering the active compound deep into the skin using lipid nanoparticles would be obtained by just using glyceryl monostearate. Thus, monoglycerides have been identified as more useful in terms of epidermal use. These observations corresponded with the results obtained by Abdelbar’s team, who also based their study on LNs containing Imwitor^®^, Compritol^®^ and Witepsol^®^ —analogous sizes of the resulting carrier structures were noted [24]. Satisfactory results for SLN production using glyceryl monostearate were also generated by Prof. Müller’s research group, which confirmed the effectiveness of the synthesis of SLNs occurring within the limits of 60 °C [25]. Moreover, the reason for this can be attributed to the fact that Imwitor^®^ 900 K has a lower melting point [15], which will translate into the compatibility of the obtained dispersion in terms of cosmetic/skin surface application. It should also be said that despite Compritol^®^’s compatibility with retinol, this solid lipid was rejected due to its too high melting point, which may affect retinol’s stability [26]. Nevertheless, it should be taken into account that the nature of the lipid matrix also depends on the amounts and types of lipids and supporting emulsifiers selected [27].

#### 2.1.2. Addition of Phosphatidylcholine as a Stabilizing Agent

**Figure 1:**
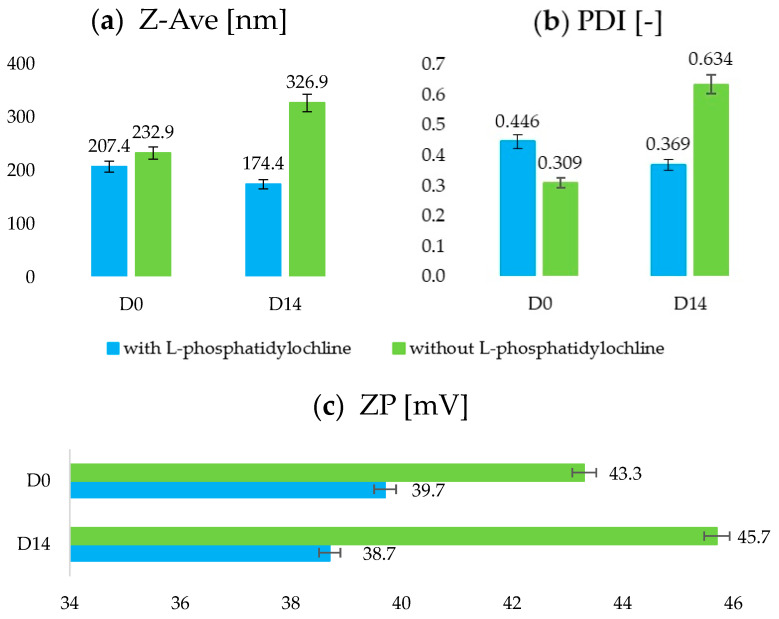
Comparison of physicochemical parameters: Z-Ave (**a**), PDl (**b**), and ZP (**c**) of dispersions of lipid nanoparticles containing and not containing phosphatidylcholine; the pHs of the solutions were within the range 5.33–5.47.

The parameters obtained for the dispersion containing no phosphatidylcholine were compared with those of the sample containing this substance (0.25 wt.%) and the solid lipid Imwitor^®^ 900 K. The comparison was carried out due to the analogous composition and the identical production method. The only element differentiating the compared formulations was the presence or absence of the aforementioned raw material (Figure 1). Testing of the physicochemical parameters was carried out on the day that the sample was made (D0) and after 14 days of storage (D14).

For the sample containing no phosphatidylcholine, the following values of physicochemical parameters (D0) were obtained: Z-Ave = 232.9 ± 2.8 nm; PDl = 0.309 ± 0.016 [−]; ZP = 43.3 ± 0.2 mV. After 14 days, there was a noticeable increase in the values of the studied parameters to the following: Z-Ave = 326.9 ± 7.4 nm (+40%); PDl = 0.634 ± 0.087 [−] (+105%); ZP = 35.7 ± 0.1 mV (+6%). It was noted that for the dispersion containing phosphatidylcholine, much more satisfactory parameter values were obtained compared to the results observed for formulations without this raw material. Moreover, a stabilization of the values of the determined parameters of the dispersion over time could be observed for the sample in which phosphatidylcholine was included. Similar observations were reported in the Molet-Rodriguez study—the increased stability of W/O/W emulsions was noted when lecithin was used in their formulation [28]. In contrast, in the case of a sample without phosphatidylcholine, a significant increase in the values of individual parameters and a decrease in the quality of the dispersion over time were noticeable. Despite comparable initial parameters, the measurement made after 14 days verified the necessity of using phosphatidylcholine in an amount of 0.25 wt.% in the final formulation. Phosphatidylcholine, acting as a stabilizing agent, does not exhibit irritant properties, which is important for the use of optimized dispersion in cosmetic formulation [29]. It excels in processes where lower temperatures are required to protect the thermal degradation of the incorporated biologically active compound [30]. Therefore, Vanić [31] believes that the presence of phospholipids (in this case, phosphatidylcholine) in the matrix of a lipid nanoparticle enhances its ability to penetrate, and thus it can help to improve the activity of the incorporated active ingredient.

#### 2.1.3. Selection of the Surfactant System

To select the appropriate surfactant system, five samples of lipid nanoparticle dispersions were made, and their physicochemical parameters were determined on the day of production of the dispersion (D0) and after 14 days of storage at room temperature (D14). The obtained results are presented in Table 2. 

When analyzing the results obtained in D0, it was noted that in samples 1–4 (containing CTAB), the Z-Ave parameter, reflecting the particle size of the dispersion, obtained satisfactory values < 300 nm. In contrast, the value of the mean particle size determined for sample No. 5 significantly exceeded the desired size range. On this basis, the variant of the dispersion not containing CTAB was not repeated anymore, but the substance was left in the final dispersion formulation at 0.5 wt.%. When considering PDI, it was noted that for samples No. 2 (containing Tween^®^ 80 and CTAB) and No. 3 (with Lutrol^®^ F68, sodium cholate, and CTAB), PDI was significantly higher than in the other samples. Elevated PDl values were noted in both D0 and D14. The most promising, considering the analysis of physicochemical parameters, were sample No. 1, containing Tween^®^ 80, sodium cholate, and CTAB, and sample No. 4, with Lutrol^®^ F68 and CTAB present in the formulation. The results obtained at D0 were analyzed and then compared with those determined at D14. For both samples, the Z-Ave value remained similar (comparing measurements from D0 and D14), and any difference was within the statistical error. A more significant change was observed in the PDl values—sample 1 showed a 25% decrease in the PDI value from the baseline, while for sample 4, the polydispersity index value remained at a similar level. The zeta potential results for all samples, both at D0 and at D14, remained at the desired level of >|±30 mV|. At values higher than |±30 mV|, the system was considered physically stable [32]. According to Dabrowska et al. [33], high and positive zeta potential values were influenced by the presence of the surfactant CTAB and its cationic nature. The negatively charged skin surface and positively charged lipid nanoparticles can interact electrostatically with each other and affect the increased permeation of active ingredients through the different layers of the epidermis [34]. 

Based on the analysis of the results additionally correlated with the available literature, the optimal surfactant system was selected: Tween^®^ 80 (1.5 wt.%) and sodium cholate (0.1 wt.%). The necessity of using CTAB (0.5 wt.%) as a component of the lipid phase of the optimized dispersion was also confirmed.

Regarding the optimization discussed, the final composition and methodology for obtaining solid lipid nanoparticles incorporated with retinol and pentapeptide were established. As a result of the numerous trials and verification of physicochemical parameters, Imwitor^®^ 900 K was included as a solid lipid in the composition of the SLN dispersion (based on the lipid screening performed). Subsequently, it was decided that L-phosphatidylcholine should be added due to the need to ensure the sufficient stability of the designed dispersion. As a last step, the surfactant system was verified—the one composed of CTAB, Tween^®^ 80 and sodium cholate was selected. The final step was the addition of active substances to the dispersion—pentapeptide and retinol—in amounts that complied with current regulations for the cosmetics industry. The qualitative–quantitative composition of the SLN dispersion thus established the starting point for its use as a component of selected cosmetic products.

### 2.2. Assessment of Encapsulation Efficiency and Loading Capacity of Retinol and Oligopeptide

Encapsulation efficiency (EE) and loading capacity (LC) are key parameters for the physicochemical characterization of lipid nanoparticles, usually determined by spectrophotometric or chromatographic methods. EE is defined as the amount of active compound that has been incorporated into lipid nanoparticles, relative to the real amount of active substance used during the production of lipid nanocarriers. LC, on the other hand, expresses the ratio of the amount of the loaded active ingredient to the total weight of the nanoparticle’s lipid components, through which it may indirectly characterize the matching of the lipid system used with the active substance [35]. The value of EE and LC is directly proportional to the lipophilicity of the incorporated raw material—for lipophilic substances, EE is usually in the 90–98% range, while hydrophilic compounds tend to reach relatively lower values due to their weak affinity for the lipid matrix [36]. Since LC determines the percentage of the active substance that can be bound to the lipid phase, this parameter for water-soluble substances will also assume lower values compared to LC values determined for lipid-soluble ingredients. 

The encapsulation efficiency of retinol (91.9 ± 0.3%), as well as the loading capacity (52.5 ± 0.1%) determined for that compound, were definitely within the expected ranges of values typical for compounds of lipophilic nature. The high affinity of retinol to the lipid structures of the obtained carriers greatly facilitated the incorporation of this compound into their matrix. It is also worth noting that the high LC value of more than 50% directly confirmed the correct selection of the quantitative ratio of retinol relative to the components of the lipid matrix of the nanoparticles. These results can be considered satisfactory, especially when comparing them with the works of other authors on the production of retinol-containing lipid nanocarriers. For example, Boskabadi and co-workers [37] reported much lower EE values—at a maximum of 66%—by encapsulating retinol into SLNs using the ultrasound technique. For the second active ingredient—pentapeptide-18—the following results were indicated: EE (75.7 ± 0.4%) and LC (21.6 ± 0.1%) significantly exceeded the values usually obtained for water-soluble compounds, which could be related to the method used to produce solid lipid nanoparticles based on W/O/W multiple emulsion. It should be mentioned that in this method, the active substances are incorporated into the internal aqueous phase, which highly reduces their undesirable penetration into the outer aqueous phase of the surfactant, which in turn could adversely affect the level of encapsulation efficiency. The EE and LC values (determined for the tested active substances) confirmed the usefulness of the high shear homogenization based on multiple emulsions for the efficient incorporation of cosmetic raw materials of both a hydrophilic and hydrophobic nature in the case of the described study of retinol and pentapeptide-18.

### 2.3. Evaluation of Stability 

The evaluation of the stability of lipid nanoparticle dispersions was necessary because the dispersion would eventually become one of the components of the cosmetic formulation. The choice of elevated temperature conditions (45 °C) was related to the aging tests of the cosmetic product, which were carried out in the following stages of this study [33]. The results obtained for the analyzed dispersions are shown in Table 3.

**Figure 2:**
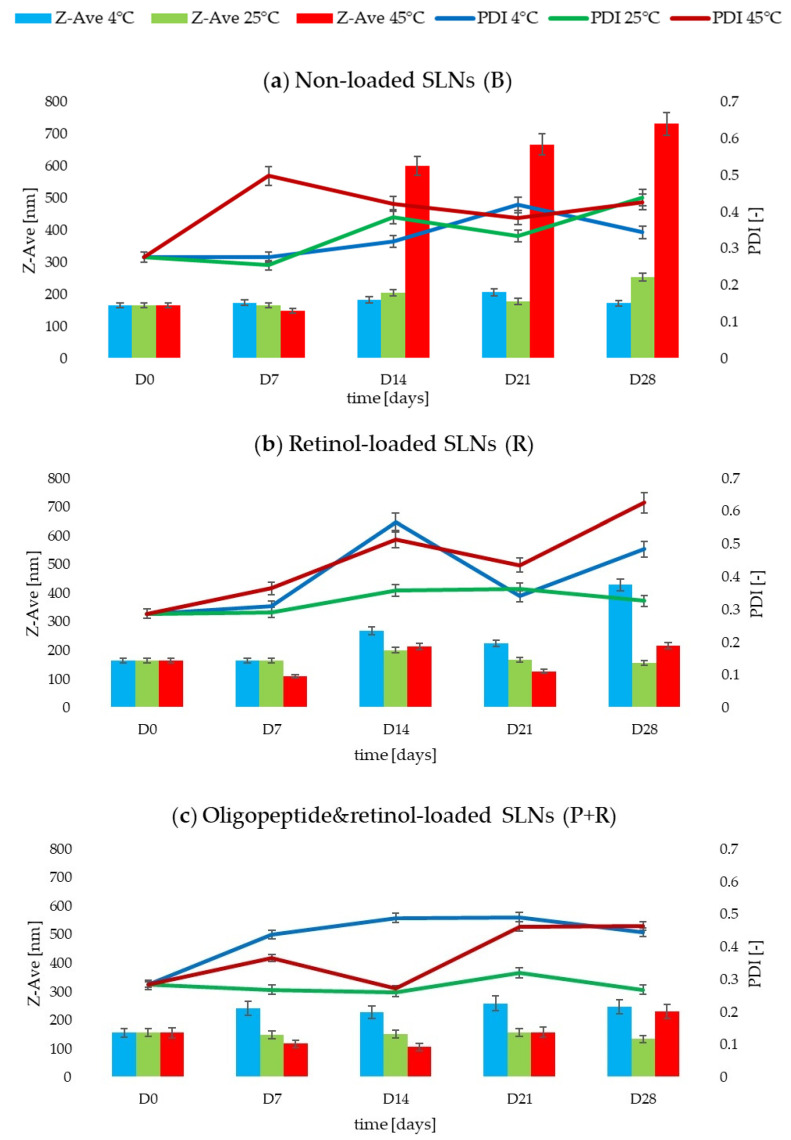
A comparison of the physicochemical parameters—Z-Ave, PDl, and ZP—of the lipid nanoparticle dispersions—B—blank (**a**); R—retinol (**b**); P+R—oligopeptide and retinol (**c**)—stored under different temperature conditions (4, 25, 45 °C) for 28 days.

It was concluded that the most favorable results of physicochemical parameters (in terms of Z-Ave and PDI) were obtained for sample 3, in which oligopeptide and retinol were present. In addition, it was noted that regardless of the presence of active substances, the samples retained the best parameters at 25 °C. Analysis of the data for individual samples stored for 28 days at different temperatures yielded the following observations:

- The changes in the value of the mean particle size of the dispersion depended on the presence of the active substances. For non-incorporated lipid nanoparticles (B), over the assumed 28 days, the Z-Ave value increased depending on the storage temperature by 4% at 4 °C, by 35% at 25 °C and by 77% at 45 °C (*p* < 0.05) (Figure 2a). The retinol-loaded SLNs (R) recorded Z-Ave values 62% (4 °C) and 24% (45 °C) higher (*p* < 0.05) compared to the results obtained at D0 and D28. On the other hand, at 25 °C, there was a 4% decrease in the value from 164.0 ± 0.5 to 157.9 ± 1.6 nm (Figure 2b). For sample 3 (P+R) enriched with two active substances, the following values of Z-Ave at day 28 were obtained: 248.3 ± 7.4 nm (+37%), 134.7 ± 0.3 nm (−16%) and 231.1 ± 1.4 nm (+32%) for storage temperatures of 4, 25 and 45 °C, respectively (*p* < 0.05) (Figure 2c). Significantly, despite the noticeable changes in Z-Ave values for dispersion 3, regardless of storage conditions, the value of 300 nm indicative of stable dispersion in this case was not exceeded [38].
- In the case of the polydispersity index (PDI), an increase in the value of this parameter was noted over time and under different storage conditions of the samples. The most noticeable changes in PDI values were noted for samples 1 (B) and 2 (R) stored at 45 °C for 28 days, changing by +35% and +54%, respectively (*p* < 0.05) (Figure 2a,b). Sample 3 (P+R) showed 36% and 39% increases in PDI at 4 °C and 45 °C, respectively (*p* < 0.05). The exception was sample 3, stored at 25 °C, for which the polydispersity index value decreased slightly (by 6%) from 0.284 ± 0.002 [−] (D0) to 0.269 ± 0.017 [−] (D28, *p* < 0.05) (Figure 2c). The PDI value for this sample did not exceed the value of 0.3 [-], considered in this study to be a value representing a stable dispersion [38].
- There were no statistically significant changes in the zeta potential values of the samples tested. There was also no correlation between the presence or absence of bioactive substances and variable storage conditions and the ZP results. In all three cases (B, R, P+R), the values were above the desired value of +30 mV, both at D0 and D28. After 28 days of stability testing, the parameters of sample 1 (B) stored at the three assumed temperatures were in the range 38.6 ± 0.4–44.6 ± 1.3 mV. The zeta potential values for sample 2 (R) were 39.9 ± 0.4 mV, 53.5 ± 5.2 mV, and 45.5 ± 0.4 mV (D28) for temperatures of 4, 25, and 45 °C, respectively. In contrast, the ZP results for sample 3 (P+R) oscillated between 42.7 ± 1.2 and 44.5 ± 1.6 mV (D28). It was shown that the incorporation of active substances (oligopeptide and retinol) did not affect the ZP values determined for the samples of lipid nanoparticle dispersions. The cationic surfactant (CTAB), present in the composition of the dispersion, was primarily responsible for the positive zeta potential value [39].
- A comparison of the tested samples (B, R, P+R) containing different systems of encapsulated active substances indicated that the presence of these compounds influenced smaller variations in the values of parameters related to dispersion stability. This was, for example, evident by comparing the Z-Ave results obtained for samples stored at 25 °C, where sample 1(B) showed a 35% increase in mean particle size, while sample 2 (R) achieved an increase of 4%, and sample 3 (P+R) showed a 16% decrease in Z-Ave.

In the course of stability testing, the following ranges of values of physicochemical parameters were obtained for the lipid nanoparticle dispersion containing oligopeptide and retinol: Z-Ave: 134.7 ± 0.3–248.3 ± 7.4 nm; PDl: 0.269 ± 0.017–0.464 ± 0.006 [−]; ZP: 42.7 ± 1.2–44.5 ± 1.6 mV. The results were obtained after 28 days of storage under three different temperature conditions (4, 25, 45 °C). The storage conditions recommended for the designed dispersion were found to be room temperature, and the slight variations in temperature conditions that occur should not affect the stability of the dispersion. The results were determined to be favorable for the use of SLN as a raw material in cosmetic formulations, where highly stable lipid carriers are necessary [18,40].

### 2.4. Characterization of Lipid Matrices

The solid state of the lipid nanoparticles and the procedure for the preparation of SLN-type lipid nanoparticles were confirmed by DSC and XRD techniques. For developed SLNs, melting peak values decreased compared to bulk Imwitor^®^ 900 K (65.4 °C), but still, all three dispersions (B; R; P+R) showed onset and melting points higher than 40 °C, which is a prerequisite when it comes to designing lipid nanoparticles for dermal application [41]. Table 4 shows the DSC parameters, including melting point, onset and enthalpy, for all the tested samples.

Lipid nanoparticles are classified as crystalline materials; hence, their melting behavior and polymorphic form can be characterized using the DSC method. Imwitor^®^ 900 K, used for the production of lipid nanoparticles, is always represented on the thermogram in the form of a single peak with a maximum at about 65 °C. Hence, in bulk form, it mainly occurs in the stable β form. The process of formation of the lipid nanoparticles is associated with the transition of the bulk lipid to the colloidal state through the β’ form but back to the α form, which is usually reflected as a shift in the peak maximum toward lower values. By analyzing the DSC parameters, this manifested, as mentioned above, as a decrease in the melting point and onset temperature of dispersion of lipid nanoparticles by about 3–4 °C for SLNs, both non-loaded and loaded with bioactive components. The similar melting behavior for the dispersions tested (B; R; P+R) confirmed the effective incorporation of retinol and oligopeptide into the lipid nanoparticles not affecting the polymorphic form and stability of the dispersion system. Since the decrease in the values of the DSC parameters shown in Table 4, relative to bulk Imwitor^®^, was not significant, the presumed desirable type of SLN-type lipid nanoparticles was confirmed. In contrast, a much more pronounced decrease in the values of these parameters is usually observed with NLCs (nanostructured lipid carriers). For example, in the work of Averina et al. where liquid lipid (*Pinus sibirica* seed oil) was added to the lipid matrix, the decrease in melting point and onset temperature relative to solid lipid were significantly sizable, which may have resulted in the formation of lipid nanostructures with lower stabilities [42].

**Figure 3:**
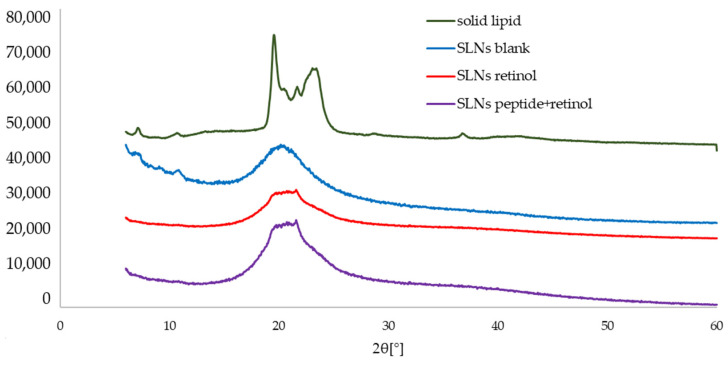
A comparison of the X-ray diffraction patterns of the solid lipid and the studied dispersions of SLNs (the diffractograms are shifted by a constant value of 14,000 arbitrary units in relation to the previous diffractogram).

The DSC data were confirmed by XRD analysis. Figure 3 shows the diffractograms for bulk Imwitor^®^ 900 K and unloaded and loaded SLN dispersions. The crystallization profile of the solid lipid revealed two scattered peaks at the angles 2Θ = 19.5° and 23.2°, which indicated the presence of the metastable polymorphic form β′. The effect of the SLN production process itself on the polymorphic state of the lipid was analyzed for both non-incorporated and incorporated formulations. From the patterns, it was noticed that for all three SLN dispersions, the β form was no longer determined. Instead, the observed characteristic single peak on the diffractograms of the dispersions of the lipid nanoparticles was studied. It was attributed to the α form in which the lipid-forming lipid nanoparticle was found due to the transition to a higher energy level after the production of LNs. Analogous results were obtained by Silva et al. when working on SLNs also based on Imwitor^®^ 900 K as a solid lipid [43]. Despite the use of different methods for lipid nanoparticle production, the DSC parameters and crystallization profiles did not differ significantly from those determined for the nanocarriers developed within the framework of this paper. Thus, it can be concluded that the melting behavior of lipid nanoparticles depends mainly on the solid lipid used in their production and the polymorphic form, which affects the values of the determined DSC parameters and XRD results.

### 2.5. Assessment of the Effect of Cosmetic Products on Selected Skin Parameters

**Figure 4:**
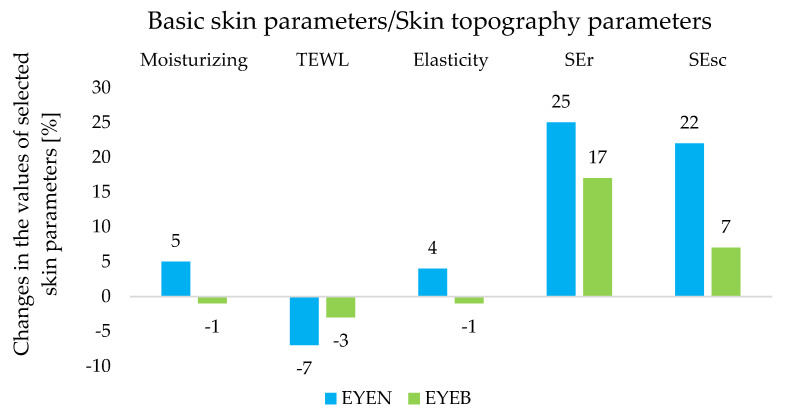
Changes in the values of basic skin parameters and selected skin topography parameters determined during an in vivo study for night eye creams after 8 weeks of application.

Skin’s hydration is one of the main parameters determining its proper functioning and healthy appearance. Numerous biochemical processes depend on the condition of the epidermis and on its barrier properties [44], and symptoms such as roughness and scaliness are the result of insufficient hydration [45]. The ability of the epidermis to retain water is determined by the condition of the protective barrier. The degree of hydration is closely related to the water content within the dermis. As a result of the difference in osmotic pressure, water passes through successive layers of the epidermis and evaporates into the environment [46]. This process is called transepidermal water loss (TEWL) and is a natural process that occurs within the structures of the skin [47]. The EYEN cream, containing SLNs incorporated with retinol and oligopeptide, showed stronger moisturizing properties compared to the EYEB cream, which is only a base without lipid nanoparticles. The results are shown in Figure 4. After 8 weeks of using EYEN, the degree of skin hydration in the eye area increased by 5% (*p* < 0.05) compared to baseline (W0), while measurements made for the cosmetic base (EYEB) showed changes in hydration at a statistically insignificant level. These results corresponded with the values of the TEWL parameter—there was a 7% decrease for the EYEN product and a 3% decrease for EYEB (*p* < 0.05). The noticeable difference in the obtained values of the degree of skin hydration may have been related to the presence of solid lipid nanoparticles, which, exhibiting occlusive properties, may have influenced the reduction in water evaporation from the epidermis [5].

The analysis of SEr, a parameter characterizing skin roughness, and SEsc, a parameter of scaliness, is also important for the diagnosis of epidermal conditions and the degree of keratolytic processes (Figure 4). The SEr value recorded after 8 weeks of EYEN application indicated an increase in the degree of roughness by 25%, while for EYEB, the increase was 17% (*p* < 0.05) compared to the results obtained at W0. According to the manufacturer’s instructions, the lower the SEr value, the rougher the skin [48]. In addition, the SEsc parameter for the EYEN product increased its value by 22% (T0→T8), while for EYEB, it increased by only 7% (*p* < 0.05). The lower the SEsc value, the less desquamation of the stratum corneum occurred, indicating a higher level of skin hydration [48]. The analyzed results testified to an accelerated process of the desquamation of the stratum corneum and its increased degree (for EYEN), while these phenomena could also affect the roughness of the epidermal surface. Certainly, the desquamation was related to retinol contained in SLNs, which actively affects the skin by accelerating cell renewal and increasing proliferation while, at the same time, enhancing the differentiation of keratinocytes and synthesis of collagens I, III, and VII [49,50]. Importantly, however, it had little effect on hydration parameters and TEWL, the results of which are described above.

Another important parameter was also analyzed, namely skin elasticity, reflecting the anti-aging effectiveness of the cosmetic product. Measurements of the flabbiness and deformability of the skin in the eye area showed that after an 8-week application of the EYEN cream, the value of the parameter increased by 4% (*p* < 0.05), while in the case of the EYEB product, it decreased by 1% with respect to the results from the day of the study, which was considered a statistically insignificant change (Figure 4). The anti-aging properties of the EYEN product containing SLN with active substances (retinol and peptide) were unequivocally confirmed. Satisfactory elasticity values may also have been influenced by proper epidermal hydration. Dry and dehydrated skin tends to be less elastic and more rigid compared to skin characterized by an adequate degree of hydration [46].

**Figure 5:**
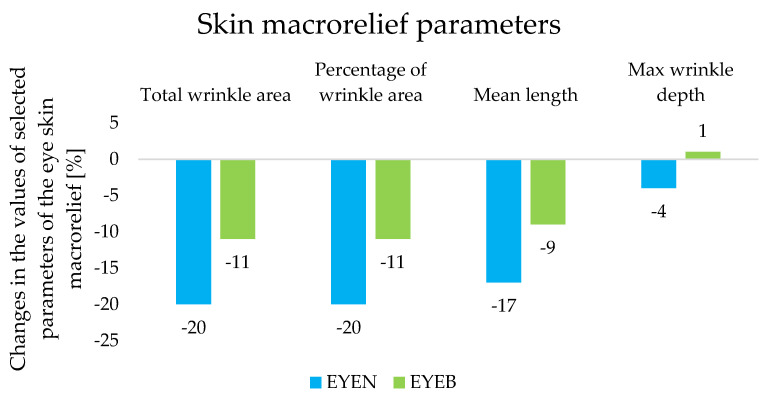
Changes in the values of skin macrorelief parameters determined during an in vivo study for night eye creams after 8 weeks of application.

Analysis of skin macrorelief images using the profilometric method made it possible to assess the number, depth, and length of wrinkles present on the characterized skin surface in the eye area. The results of this study are summarized in Figure 5. After 8 weeks of using EYEN and EYEB creams, the total wrinkle area was reduced by 20% and 11%, respectively (*p* < 0.05). Moreover, there was also a reduction in the mean length and maximum depth of wrinkles when the EYEN preparation was applied. Mean length was shown to be reduced by 17% and maximum wrinkle depth by 4% (*p* < 0.05). On the other hand, in the case of the cosmetic base (EYEB), there was a 9% decrease in the value corresponding to the length of wrinkles (*p* < 0.05) and a 1% increase in their maximum depth, with the magnitude being within the statistical error; hence, our conclusion was that there was no improvement in this parameter for EYEB.

The results obtained after an 8-week application of the tested cosmetic formulations undoubtedly indicated an improvement in the appearance of the skin and a reduction in the appearance of lines and wrinkles on its surface. Hence, the anti-wrinkle properties of the EYEN cosmetic product containing solid lipid nanoparticles encapsulated with retinol and oligopeptide were confirmed. The effectiveness of retinol-loaded lipid nanoparticles was also confirmed by Ferreira et al. They noted lower irritation potential and a lower degree of skin peeling of SLNs enriched with retinol compared to the use of retinoid in its classic form [6]. Salvioni et al., on the other hand, mentioned the occlusive properties of SLNs, which had an influence on the moisture level of the skin [29].

It should be mentioned here that the skin around the eyes is very thin (about 0.35 mm on the eyelids) [45], which makes its susceptible to loss of elasticity and hydration, and thus the formation of wrinkles relatively common [51]. Based on the in vivo study, it was concluded that the properties of the EYEN cream can fully meet the needs of the delicate skin around the eyes.

## 3. Materials and Methods

### 3.1. Materials

Four different solid lipids were selected for lipid screening—Compritol^®^ 888 ATO (glyceryl dibehenate), Precirol^®^ ATO 5 (glyceryl distearate) from Gattefossé (Lyon, France), Imwitor^®^ 900 K (glyceryl monostearate) and Witepsol^®^ H15 (a blend of tri-, di- and monoglycerides of fatty acids) from IOI Oleo GmbH (Hamburg, Germany). The dispersions of the lipid nanoparticles were produced by using glycerol (99.5%; Chempur, Piekary Śląskie, Poland), CTAB (hexadecyltrimethylammonium bromide, ≥98%; Chemat, Gdańsk, Poland), L-phosphatidylcholine (from soybean, Type II-S, 14–29% choline basis) and Lutrol^®^ F68 (>90%) from Sigma-Aldrich (Steinheim, Germany), as well as sodium cholate (99%; Acros Organics, Antwerp, Belgium) and Tween^®^ 80 (polysorbate 80; Pol-Aura, Zawroty, Poland). The incorporated active compounds—Retinol GS50 (retinol, 50%) and Leuphasyl^®^ peptide (pentapeptide-18/Tyr-D-Ala-Gly-Phe-Leu, 99.5%)—were purchased from DSM (Kaiseraugst, Switzerland) and Lipotec (Barcelona, Spain), respectively.

### 3.2. Optimization of the Composition of Lipid Nanoparticles—Selected Factors

The optimization of the composition of the lipid nanoparticles started with the selection of a retinol-compatible solid lipid, as the composition of the lipid matrix is crucial for the stability of the SLN [23]. First, appropriate amounts of pre-selected solid lipids and retinol were weighed into glass vials. The components were then heated to a temperature above the melting point of the solid lipid. In the final step, the resulting mixtures were observed at 15 min, 30 min, 1 h, 24 h, and 72 h after the system was solidified. The experiment aimed to obtain a compatible and homogeneous mixture from the combination of retinol and solid lipid. Four solid lipids were used in this study: Compritol^®^ 888 ATO (glyceryl dibehenate), Precirol^®^ ATO 5 (glyceryl distearate), Imwitor^®^ 900 K (glyceryl monostearate) and Witepsol^®^ H15 (a blend of tri-, di- and monoglycerides of fatty acids). Solid lipids found to be compatible with the tested active ingredient after 72h were subjected to further testing as components (1.0 wt.%) of lipid nanoparticle dispersions. 

The next optimization step was to verify the necessity of including phosphatidylcholine in the dispersion. This compound belongs to the group of glycerophospholipids (the most common polar lipid compounds found in nature), mainly in the outer parts of the cell membrane [52]. Phosphatidylcholine is also credited with the ability to ensure the stability of the lipid nanoparticle dispersions containing it [53]. In the course of this study, lipid nanoparticles differing regarding the presence (0.25 wt.%) or absence of the raw material were compared.

A combination of four different surfactants was also used during optimization. Included in this study were surfactants belonging to the non-ionic group—Tween^®^ 80 (polysorbate 80) and Lutrol^®^ F68 (polyoxyethylene–polyoxypropylene block copolymer), anionic–sodium cholate, and cationic-hexadecyltrimethylammonium bromide (CTAB). Optimization at the surfactant system selection stage consisted solely of varying the composition of the external aqueous phase for producing the target lipid nanoparticles. Various surfactant systems were tested based on a combination of 1.5 wt.% non-ionic surfactant with or without 0.1 wt.% sodium cholate while assuming a constant CTAB content of 0.5 wt.%. One trial was also performed, during which the use of CTAB in the lipid phase was abandoned.

### 3.3. Lipid Nanoparticles—Method of Production

Solid lipid nanoparticles were produced by applying a high shear homogenization (HSH) method based on multiple emulsion (W/O/W). The first step was to introduce the components of the external aqueous phase (I) into a glass beaker (0.1 wt.% sodium cholate and 1.5 wt.% Tween^®^ 80 (polysorbate 80) were dispersed in distilled water) and heat them to 35 °C while stirring at 250–300 rpm for 15 min. Then, the system was cooled to room temperature. At the same time, the components of the lipid phase (II)—1.0 wt.% Imwitor^®^ 900 K (glyceryl monostearate), 0.5 wt.% CTAB (hexadecyltrimethylammonium bromide) and 0.25 wt.% L-phosphatidylcholine—were introduced into a glass beaker and heated to the melting point of the solid lipid. Next, 2.0 wt.% of retinol was added to the properly heated lipid phase (II) and cooled to 60 °C. The next step was to gradually pour the heated glycerol into a beaker with the lipid phase (II) and subject the resulting mixture to continuous stirring for 10 min. At the same time, an aqueous solution of 0.1 wt.% Leuphasyl^®^ peptide (pentapeptide-18/Tyr-D-Ala-Gly-Phe-Leu), which was the internal aqueous phase (III), was prepared in a glass vial. The solution was heated to 60 °C and then added to the lipid phase (II). The key step was to subject the system to high shear homogenization (Ultra-Turrax^®^ DI 25 Basic, IKA-Werke GmbH, Staufen im Breisgau, Germany) at 8000 rpm for 15 min. The resulting W/O emulsion was combined in the final step with the external aqueous phase (I) under continuous stirring at 500 rpm for 15 min.

### 3.4. Lipid Nanoparticles—Physicochemical Parameters

The three basic physicochemical parameters of lipid nanoparticle dispersions were determined using a Zetasizer Nano ZS (Malvern Instruments, Malvern, UK). The mean particle size (Z-Ave), polydispersity index (PDI), and zeta potential (ZP) were taken into account. Measurements were performed on aqueous solutions of SLN dispersion with appropriate concentrations and pH values. The measurement procedure was repeated three times for each sample, and the arithmetic mean and standard deviation were calculated from the obtained results.

### 3.5. Encapsulation Efficiency and Loading Capacity

The encapsulation efficiency (EE) and loading capacity (LC) of retinol and oligopeptide in solid lipid nanoparticles were determined indirectly by the centrifugation technique, assessing the free retinol and pentapeptide-18 (non-incorporated) contents by UV-Vis spectroscopy (Varian Cary 50 Bio UV-Vis Spectrophotometer, Varian Inc./Agilent Technologies, Santa Clara, CA, USA). The spectroscopic method was previously validated and involved the quantification of non-encapsulated active substances present in the external aqueous phase of the surfactant of LNs dispersion. The samples were prepared by measuring 1 mL of the dispersion into an Eppendorf Tubes^®^ test tube, placing it in the angular rotor of an MPW-350R laboratory centrifuge (MPW MED. INSTRUMENTS, Warsaw, Poland), and centrifuging at 4000 rpm for 30 min. In the next step, 20 µL of the supernatant (the aqueous solution of the external phase separated by centrifugation) was taken into the flask, which was subsequently increased up to 10 mL with a 50:50 mixture of phosphate buffer (pH = 5.8) and ethanol. The sample was then shaken vigorously for 5 min. The final step was to convert the concentration results into encapsulation efficiency and loading capacity (expressed as a percentage), in accordance with the following equations [54]:

(1) $$EE\left[\%\right]=\frac{total\;amount\;of\;active\;substance-amount\;of\;non\text{-}incorporated\;active\;substance}{total\;amount\;of\;active\;substance}$$

(2) $$LC\;\left[\%\right]=\frac{total\;amount\;of\;active\;substance-amount\;of\;non\text{-}incorporated\;active\;substance}{total\;amount\;of\;lipid}$$

### 3.6. Stability Test

The confirmation of the stability of the lipid nanoparticle dispersions was carried out by verifying changes in the values of three parameters (Z-Ave, PDI, and ZP; Zetasizer Nano ZS instrument (Malvern Instruments, Malvern, UK) over time and under varying temperature conditions. The stability test was performed for the target optimized dispersions of SLNs: sample 1 (B, blank)—non-incorporated lipid nanoparticles; sample 2 (R)—SLNs containing 2.0 wt.% of retinol; sample 3 (P+R)—SLNs loaded with 0.1 wt.% of pentapeptide-18 and 2.0 wt.% of retinol. The experiment was conducted on the day that the lipid nanoparticles were received (D0) and after 7, 14, 21, and 28 days of storage at 4, 25, and 45 °C. The statistical analysis was carried out using Statistica 10.0 software. The level of statistical significance was set at *p* < 0.05.

### 3.7. X-ray Diffraction (XRD) and Differential Scanning Calorimetry (DSC) Analysis

The characterization of the lipid matrices of SLNs was performed by using X-ray diffraction (XRD; D8 Advance powder diffractometer combined with a Johansson monochromator/Bruker, Billerica, MA, USA) and differential scanning calorimetry (DSC; DSC 8500 differential scanning calorimeter/PerkinElmer, Waltham, MA, USA). Both samples of solid lipid and dispersions of lipid nanoparticles were analyzed. The procedure of sample preparation and the detailed conditions of DSC and XRD analysis for lipid nanoparticle dispersions were described in earlier papers [55]. In brief, the principle of DSC measurement was to gradually heat a sample of solid lipid or SLN dispersions from 25 °C to 90 °C in a flow of nitrogen (20 mL/min) at a scanning rate of 5 °C per minute, maintaining the sample at 90 °C for 1 min, and then cooling it to 25 °C with similar parameters. On the other hand, during XRD analysis, samples were dried at room temperature before measurement. For each sample, the procedure was carried out within the high-angle range (2Θ = 6.0–60.0°).

### 3.8. Cosmetic Products/Eye Creams—Preparation 

This study was performed on two cosmetic preparations—two night eye creams produced by Dottore Polska Sp. z o.o., Poznan, Poland. The formulation of the cosmetic products was in accordance with Regulation (EC) No 1223/2009/EC of the European Parliament and the Council of 30 November 2009 on cosmetic products with subsequent updates. One of the cosmetic formulations contained a tested SLN dispersion, which was added in the last technological stage with continuous mixing to ensure the uniform consistency of the cosmetic mass. The exact quantitative composition and manufacturing technology of the eye creams remain the sole knowledge of Dottore Polska Sp. z o.o. Two cosmetic products of analogous composition (O/W emulsions with 24.5 wt.% of lipid phase), differing only in the presence of lipid nanoparticle dispersion, were used in a comparative study, which is discussed within the scope of this paper. The INCI compositions of night eye creams investigated are shown below.

#### 3.8.1. EYEN (Anti-Aging Night Eye Cream Containing 1.0 wt.% of Lipid Nanoparticles Loaded with Retinol and Pentapeptide-18)

Ingredients (INCI): Aqua, Butylene Glycol, Coco-Caprylate/Caprate, Pentylene Glycol, Propanediol, Cetyl Alcohol, Glyceryl Stearate, Simmondsia Chinensis Seed Oil, Triisostearin, Glyceryl Stearate Citrate, Butyrospermum Parkii Butter, Theobroma Cacao Seed Butter, Glycerin, Cetyl Ricinoleate, Persea Gratissima Oil, retinol, Sodium Hyaluronate, Acetyl Tetrapeptide-5, Palmitoyl Tripeptide-5, Palmitoyl Dipeptide-5 Diaminobutyroyl Hydroxythreonine, Tetradecyl Aminobutyroylvalylaminobutyric Urea Trifluoroacetate, Pentapeptide-18, Phosphatidylcholine, Cetrimonium Bromide, sodium cholate, Potassium Cocoyl Hydrolyzed Rice Protein, Sodium Cocoyl Amino Acids, Caprylic/Capric Triglyceride, Tocopheryl Acetate, Tocopherol, Helianthus Annuus Seed Oil, Raspberry Ketone, Stearyl Alcohol, Polysorbate 80, Xanthan Gum, Magnesium Chloride, Tetrasodium Glutamate Diacetate, Sodium Hydroxide, Trisodium NTA, and Parfum.

#### 3.8.2. EYEB (Anti-Aging Night Eye Cream without Lipid Nanoparticles)

Ingredients (INCI): Aqua, Butylene Glycol, Coco-Caprylate/Caprate, Pentylene Glycol, Propanediol, Cetyl Alcohol, Glyceryl Stearate, Simmondsia Chinensis Seed Oil, Triisostearin, Glyceryl Stearate Citrate, Butyrospermum Parkii Butter, Theobroma Cacao Seed Butter, Glycerin, Cetyl Ricinoleate, Persea Gratissima Oil, Sodium Hyaluronate, Acetyl Tetrapeptide-5, Palmitoyl Tripeptide-5, Palmitoyl Dipeptide-5 Diaminobutyroyl Hydroxythreonine, Tetradecyl Aminobutyroylvalylaminobutyric Urea Trifluoroacetate, Potassium Cocoyl Hydrolyzed Rice Protein, Sodium Cocoyl Amino Acids, Caprylic/Capric Triglyceride, Tocopheryl Acetate, Tocopherol, Helianthus Annuus Seed Oil, Raspberry Ketone, Stearyl Alcohol, Xanthan Gum, Magnesium Chloride, Tetrasodium Glutamate Diacetate, Sodium Hydroxide, Trisodium NTA, and Parfum.

### 3.9. Evaluation of the Effectiveness of Cosmetic Products—In Vivo Study

Application tests were carried out on a group of 20 female volunteers aged 30–65 years old with signs of aging in the eye area, such as wrinkles and skin sagging. Before this study, the volunteers were informed about the implications of this study (in the form of information material) and signed an informed consent form to participate in the tests. Each participant received two cosmetic products: EYEN (an eye cream containing lipid nanoparticles) and EYEB (a formulation devoid of active substances encapsulated in lipid carriers). The cosmetic formulations tested were to be applied once a day at night. During this time, the application of cosmetics of similar or analogous compositions and actions was prohibited. Sun exposure, including tanning beds, was also to be avoided (due to the presence of retinol in the cream formulation). Any adverse effects were to be reported to the principal investigator.

The duration of the in vivo study was 8 weeks and approved by the Bioethical Commission of Poznan University of Medical Sciences, Poland, on 12 October 2023 (768/23). Skin parameters were measured before the application of the products on the day that this study started (W0) and after 8 weeks (W8) of use. To assess skin condition, the following equipment from Courage + Khazaka electronic GmbH (Cologne, Germany) was used: Tewameter^®^ TM 300 (transepidermal water loss level, TEWL); Corneometer^®^ CM 825 (epidermal hydration level); Cutometer^®^ MPA 580 (skin elasticity); Visioscan^®^ VC 98 (skin topography parameters); Visioline^®^ VL 650 (skin macrorelief parameters). The Wilcoxon test for pairs of observation results was used for statistical analysis. The level of significance was *p* < 0.05.

## 4. Conclusions

The use of solid lipid nanoparticles encapsulated with retinol and pentapeptide (Tyr-D-Ala-Gly-Phe-Leu) in the cosmetic industry is perfectly in line with the trend of skin minimalism. The main advantage of using lipid nanocarriers is the provision of assisted transepidermal transport, which enables the delivery of active substances with prolonged action directly to the deeper layers of the epidermis. The additive nature of the active compounds incorporated into SLNs provides a broader spectrum of action and offers more functions than retinol and peptide used in therapy alone. Through numerous trials to optimize the SLN formulation, it was proven that Imwitor^®^ 900 K was most compatible as a solid lipid. The presence of L-phosphatidylcholine as a polar lipid compound also proved crucial for dispersion. In contrast, the surfactant group represented by the combination of Tween^®^ 80, sodium cholate, and CTAB proved to be the most effective. The physicochemical parameters of the designed lipid nanoparticles enriched with retinol and oligopeptide, such as a particle size (Z-Ave) of less than 200 nm, a polydispersity index (PDI) of up to 0.3, and a zeta potential (ZP) of >30 mV, met the requirements of their functionality for cosmetic application. The efficiency of transepidermal transport (using SLNs) was confirmed by in vivo studies, indicating the potential of these lipid nanoparticles as a cosmetic raw material in terms of reducing wrinkles and supporting the regeneration of the delicate skin around the eyes. It should be mentioned that there are few cosmetic products on the cosmetic market that use lipid nanoparticle technology. Available solid lipid nanoparticles usually contain only one active ingredient, such as retinol itself. Peptides incorporated in the lipid matrix are virtually absent in cosmetics. The presented research proved the effectiveness of SLNs containing simultaneously lipophilic retinol and hydrophilic oligopeptide, which is an innovative solution not previously used in the cosmetics industry.

## Figures and Tables

**Table 1:** Results of lipid screening—observation of homogeneity of mixtures (solid lipids combined with retinol) over time.

Solid Lipid	Solubility					
	0 min	15 min	30 min	1 h	24 h	72 h
Compritol^®^ 888 ATO	+	+	+	+	+	+
Imwitor^®^ 900 K	+	+	+	+	+	+
Precirol^®^ ATO 5	−	−	−	−	−	−
Witepsol^®^ H15	+	+/−	+/−	+/−	−	−

“+” soluble; “−” insoluble.

**Table 2:** The optimization of the surfactant system—the changes in the physicochemical parameters (Z-Ave, PDl, ZP) of the tested dispersions of lipid nanoparticles over time (data are expressed as mean ± standard deviation).

	Surfactant System	Results	D0	D14
1.	Tween^®^ 80	Z-Ave [nm]	165.1 ± 0.5	173.9 ± 0.9
	sodium cholate	PDI [−]	0.379 ± 0.001	0.286 ± 0.002
	CTAB	ZP ^1^ [mV]	42.3 ± 1.3	42.7 ± 0.4
2.	Tween^®^ 80	Z-Ave [nm]	200.0 ± 0.7	185.7 ± 1.5
	−	PDI [−]	0.403 ± 0.004	0.417 ± 0.013
	CTAB	ZP ^1^ [mV]	45.6 ± 0.2	43.9 ± 2.3
3.	Lutrol^®^ F68	Z-Ave [nm]	203.5 ± 2.4	475.8 ± 19.8
	sodium cholate	PDI [−]	0.456 ± 0.012	0.489 ± 0.042
	CTAB	ZP ^1^ [mV]	43.6 ± 1.1	39.6 ± 5.2
4.	Lutrol^®^ F68	Z-Ave [nm]	192.1 ± 0.4	180.9 ± 0.5
	−	PDI [−]	0.396 ± 0.005	0.374 ± 0.019
	CTAB	ZP ^1^ [mV]	39.7 ± 0.4	38.1 ± 0.7
5.	Lutrol^®^ F68	Z-Ave [nm]	5618.0 ± 33.0	−
	sodium cholate	PDI [−]	0.380 ± 0.022	−
	−	ZP ^1^ [mV]	−	−

^1^ The pHs of the solutions are within the range 5.01–5.47.

**Table 3:** The changes in the values of the mean particle size (Z-Ave), the polydispersity index (PDl), and the zeta potential (ZP) of the lipid nanoparticle dispersions (B—blank; R—retinol; P+R—oligopeptide and retinol) stored under different temperature conditions (4, 25, 45 °C) for 28 days (data are expressed as mean ± standard deviation).

		Temperature	D0	D7	D14	D21	D28
1. (B) blank	Z-Ave [nm]	4 °C		173.5 ± 0.9	183.5 ± 0.4	206.3 ± 0.5	172.3 ± 1.1
		25 °C	165.1 ± 1.1	165.9 ± 0.4	204.5 ± 1.2	177.8 ± 0.5	253.1 ± 9.8
		45 °C		147.8 ± 0.8	600.8 ± 12.3	667.4 ± 9.8	731.6 ± 7.4
	PDl [−]	4 °C		0.276 ± 0.008	0.319 ± 0.023	0.419 ± 0.02	0.344 ± 0.007
		25 °C	0.379 ± 0.007	0.255 ± 0.006	0.386 ± 0.032	0.334 ± 0.008	0.439 ± 0.013
		45 °C		0.498 ± 0.039	0.422 ± 0.027	0.384 ± 0.023	0.427 ± 0.022
	ZP ^1^ [mV]	4 °C		43.0 ± 1.1	40.3 ± 0.4	39.5 ± 0.9	38.6 ± 0.4
		25 °C	42.7 ± 0.9	41.1 ± 0.9	42.3 ± 0.8	42.7 ± 0.8	33.1 ± 0.3
		45 °C		41.0 ± 0.6	45.0 ± 0.3	43.5 ± 0.5	44.6 ± 1.3
2. (R) retinol	Z-Ave [nm]	4 °C		163.9 ± 0.4	268.4 ± 0.5	224.8 ± 5.4	429.4 ± 12.3
		25 °C	164.0 ± 0.5	164.5 ± 1.1	202.1 ± 1.2	168.6 ± 0.3	157.9 ± 1.6
		45 °C		111.3 ± 1.4	213.4 ± 4.4	128.0 ± 0.23	216.7 ± 6.4
	PDl [−]	4 °C		0.311 ± 0.018	0.566 ± 0.022	0.341 ± 0.011	0.485 ± 0.036
		25 °C	0.287 ± 0.008	0.292 ± 0.008	0.358 ± 0.011	0.364 ± 0.015	0.327 ± 0.020
		45 °C		0.365 ± 0.005	0.515 ± 0.014	0.436 ± 0.012	0.626 ± 0.017
	ZP ^1^ [mV]	4 °C		37.6 ± 0.7	44.5 ± 0.1	38.1 ± 0.8	39.9 ± 0.4
		25 °C	43.6 ± 1.4	42.1 ± 1.2	42.6 ± 0.3	39.6 ± 1.3	53.5 ± 5.2
		45 °C		41.1 ± 0.3	43.1 ± 0.4	35.6 ± 1.1	45.5 ± 0.4
3. (P+R) oligopeptide retinol	Z-Ave [nm]	4 °C		242.3 ± 7.2	228.3 ± 1.4	259.3 ± 8.7	248.3 ± 7.4
		25 °C	156.5 ± 0.8	148.2 ± 0.8	151.8 ± 0.7	157.4 ± 1.8	134.7 ± 0.3
		45 °C		117.7 ± 0.9	107.2 ± 0.4	158.5 ± 2.9	231.1 ± 1.4
	PDl [−]	4 °C		0.438 ± 0.009	0.489 ± 0.019	0.491 ± 0.022	0.445 ± 0.022
		25 °C	0.284 ± 0.002	0.269 ± 0.006	0.261 ± 0.002	0.320 ± 0.034	0.269 ± 0.017
		45 °C		0.366 ± 0.003	0.272 ± 0.004	0.463 ± 0.007	0.464 ± 0.006
	ZP ^1^ [mV]	4 °C		40.9 ± 0.4	43.3 ± 0.5	44.5 ± 0.6	43.8 ± 0.3
		25 °C	45.6 ± 0.2	44.7 ± 0.7	45.8 ± 0.3	44.0 ± 1.9	42.7 ± 1.2
		45 °C		38.1 ± 2.1	45.1 ± 0.2	37.2 ± 0.9	44.5 ± 1.6

^1^ The pHs of the solutions within the range 4.37–5.47.

**Table 4:** DSC parameters of bulk lipid (Imwitor^®^ 900 K), non-incorporated (B), retinol-loaded (R), and oligopeptide and retinol-loaded SLNs (P+R).

Sample	Melting Peak [°C]	Onset [°C]	Enthalpy [J/g]
Imwitor^®^ 900 K	65.4	54.7	164.5
(B) blank	62.3	51.7	26.8
(R) retinol	61.6	50.7	32.1
(P+R) oligopeptide + retinol	61.7	50.9	33.6

## Data Availability

The raw data supporting the conclusions of this article will be made available by the authors upon request. The general procedure for the emulsions’ formation is only available to non-professionals.
